# Modified level of miR‐376a is associated with Parkinson's disease

**DOI:** 10.1111/jcmm.14979

**Published:** 2020-01-12

**Authors:** Masoud Baghi, Mahsa Rostamian Delavar, Elaheh Yadegari, Maryam Peymani, David Pozo, Mohammad Hossein Nasr‐Esfahani, Kamran Ghaedi

**Affiliations:** ^1^ Department of Cell and Molecular Biology and Microbiology Faculty of Biological Science and Technology University of Isfahan Isfahan Iran; ^2^ Department of Cellular Biotechnology Cell Science Research Center Royan Institute for Biotechnology Isfahan Iran; ^3^ Department of Biology Faculty of Basic Sciences Shahrekord Branch Islamic Azad University Shahrekord Iran; ^4^ CABIMER Andalusian Center for Molecular Biology and Regenerative Medicine Sevilla Spain; ^5^ Department of Medical Biochemistry, Molecular Biology and Immunology Universidad de Sevilla Sevilla Spain

**Keywords:** GSK3β, miR‐376a, mitochondrial transcription factor A, Parkinson's disease, PGC1α

## Abstract

Parkinson's disease (PD) is a frequent progressive neurodegenerative disorder. Impaired mitochondrial function is a major feature of sporadic PD. Some susceptibility or causative genes detected in PD are strongly associated with mitochondrial dysfunction including *PGC1α*, *TFAM* and *GSK3β*. microRNAs (miRNAs) are non‐coding RNAs whose altered levels are proven in disparate PD models and human brains. Therefore, the aim of this study was to detect modulations of miRs upstream of *PGC1α*, *TFAM* and *GSK3β* in association with PD onset and progress. In this study, a total of 33 PD subjects and 25 healthy volunteers were recruited. Candidate miRNA (miR‐376a) was selected through target prediction tools and literature survey. Chronic and acute in vitro PD models were created by MPP^+^‐intoxicated SHSY5Y cells. The levels of miR‐376a and aforementioned genes were assessed by RT‐qPCR. The expression of target genes was decreased in chronic model while there were dramatically up‐regulated levels of those genes in acute model of PD. miR‐376a was strongly altered in both acute and chronic PD models as well as PBMCs of PD patients. Our results also showed overexpression of *PGC1α,* and *TFAM* in PBMCs is inversely correlated with down‐regulation of miR‐376a, suggesting that miR‐376a possibly has an impact on PD pathogenesis through regulation of these genes which are involved in mitochondrial function. miR‐376a expression in PD‐derived PBMCs was also correlated with disease severity and may serve as a potential biomarker for PD diagnosis. This is the first study showing altered levels of miR‐376a in PD models and PBMCs, suggesting the probable role of this miRNA in PD pathogenesis. The present study also proposed *TFAM* and *PGC1α* as target genes of miR‐376a for the first time, through which it possibly can exert its impact on PD pathogenesis.

## 1 INTRODUCTION

Parkinson's disease (PD) is the second most frequent neurodegenerative disorder (NDD) whose pathological characteristics are progressive loss of dopaminergic neurons of midbrain and presence of intraneuronal cytoplasmic inclusions, namely “Lewy bodies”.^1^, ^2^ Around 95% of PD cases are sporadic and familial forms constitute the remainder.^3^ While the precise pathomechanism of PD is not entirely understood, some molecular mechanisms are suggested to contribute to the pathogenesis, including chronic neuroinflammation, oxidative stress, apoptosis, autophagy and mitochondrial dysfunction.^1^, ^3^, ^4^

There is no effective treatment approach to halt the development of PD currently, and its management remains symptomatic. Recently, substantial efforts have been made to comprehend molecular mechanisms responsible for PD in order to develop new therapeutic strategies for the disease.^3^, ^5^ Considering that mitochondrial dysfunction is a prominent pathological hallmark of many neurodegenerative disorders, understanding the mechanisms through which mitochondrial dysfunction takes part in the pathogenesis of these diseases can help to define novel therapeutic approaches.^6^ Thus, complex 1 inhibitors like 1‐methyl‐4‐phenylpyridinium (MPP^+^) are extensively applied as a model for mitochondrial dysfunction.^7^, ^8^ The mechanism of MPP^+^ neurotoxicity involves inducing oxidative stress, mitochondrial dysfunction and inflammation which finally lead to apoptotic cell death.^4^ Here, we served MPP^+^ as a means to reproduce PD‐related mitochondrial dysfunction in SH‐SY5Y cell line.

Notably, environmental factors as well as genetic susceptibility are decisive factors in sporadic PD aetiology.^9^ Beside environmental agents, several susceptibility or causative genes were detected in PD, some of which are strongly associated with mitochondrial dysfunction including peroxisome proliferator‐activated receptor gamma coactivator 1‐alpha (PGC1α), mitochondrial transcription factor A (TFAM) and glycogen synthase kinase 3 beta (GSK3β).^1^ PGC1α is a multifunctional coactivator of transcription factors like NRF‐1, NRF‐2, TFAM and FOXO receptors, and thereby regulates mitochondrial biogenesis, respiration as well as function.^1^, ^3^ Moreover, since PGC1α induces the expression of several reactive oxygen species (ROS) detoxifying enzymes including SOD1 and 2, catalase and glutathione peroxidase‐1, it tightly regulates oxidative capacity and reduces oxidative stress.^3^, ^10^ There is a substantial literature indicating possible links between PGC1α and diverse neurodegenerative diseases (NDDs), comprising amyotrophic lateral sclerosis (ALS), Huntington's disease (HD), Alzheimer's disease (AD) and PD.^10^ As a target gene of PGC1α, TFAM regulates mitochondrial genome replication and transcription as well as mitochondrial biogenesis.^11^ TFAM also makes a positive contribution to mtDNA stability and initiates synthesis of mtDNA‐encoded respiratory chain subunits.^7^ GSK3β is widely known for its role in the pathogenesis of a host of neurodegenerative disorders comprising PD, impacting several pathological processes such as neuronal apoptosis, neuroinflammation and dopaminergic (DA) neuron degeneration.^12^

miRNAs, a class of non‐coding RNAs, are roughly 22 bases in length and take part in a wide variety of biological and pathological processes in health and disease.^5^, ^13^ They tend to repress genes’ expression level through binding to specific sequences mostly located in the 3'‐untranslated region (3'UTR) of mRNAs, culminating in mRNA degradation or deadenylation and translation inhibition.^5^, ^14^ miRNAs have an enormous modulatory potential considering that each single miRNA can govern expression of hundreds of different transcripts and that's why whole phenotype of a disease can be feasibly affected by regulating a single miRNA molecule.^5^, ^15^, ^16^ Mounting evidence has emerged showing the association between miRNA network and pathogenesis of neurodegenerative disorders including PD.^12^ Furthermore, altered level of several miRNAs has been reported in disparate PD models and human brains of patients suffering from PD.^5^, ^12^, ^13^, ^15^, ^16^ While there is substantial literature confirming miRNAs’ implication in PD development, only a few studies have attempted to identify deregulated miRNAs.^5^, ^16^

Since maintenance of mitochondrial machinery depends on the action of some mitochondria‐related nuclear‐encoded proteins,^11^ the principal aim of this study was to assess the expression levels PGC1α, TFAM and GSK3β, as well as their predicted upstream miRNA, miR‐376a, in chronic and acute models of PD and patient's peripheral blood mononuclear cells (PBMCs).

## 2 MATERIALS AND METHODS

### 2.1 Sample collection and PBMC isolation

Our study design focused on the evaluation of disparities between PD patients and clinically healthy subjects in terms of gene expression. Therefore, a total of 33 PD subjects were recruited, attending the Parkinson's disease Assessment Unit of the Alzahra Hospital (Isfahan, Iran). The protocol of study to use human samples was confirmed by both the Bioethics Committee of University of Isfahan and ROYAN institute review board under the bioethical code number: IR.ACECR.ROYAN.REC.1397.216. Moreover, 25 healthy age‐matched volunteers without symptoms of neurological disorders or family history of PD were enrolled in the study as controls. Patients were staged based on the Hoehn and Yahr scales (HY) by an expert neurologist. Twelve patients showing unilateral involvement only usually with minimal or no functional disability (HY‐1 stage) and fourteen patients with bilateral or midline involvement without impairment of balance (HY‐2 stage) were categorized as the early‐stage PD. However, four patients suffering from mild‐to‐moderate disability with impaired postural reflexes but physically independent (HY‐3 stage) and three patients with severe disability being able to walk or stand independently without the use of an assistive device (HY‐4 stage) were regarded as the advanced stage PD.

All participators in the study signed a written informed consent. Approximately four millilitres of venous blood was collected from all subjects. Within two hours of blood collection, PBMCs were separated from whole blood using Ficoll‐Histopaque density gradient centrifugation according to the manufacturer's instruction.

### 2.2 Candidate miRNA selection

According to recent studies, three mitochondria‐related genes, including *PGC1α*, *TFAM* and *GSK3β* whose expressions were significantly altered in different neurodegenerative conditions, were taken into account to select a putative targeting miRNAs. TargetScan 7.1,^17^ online prediction software, was served to predict miRNAs which target candidate transcripts, after which Venn diagrams (http://bioinfogp.cnb.csic.es/tools/venny/index.html) were applied to compare the introduced lists of miRNAs and to choose the one with the most relation with the candidate mRNAs. Moreover, NDD‐specific signature miRNAs were identified through literature review and using HMDD version 2.0.^18^

### 2.3 Cell culture and neurotoxin treatment

Neuroblastoma SH‐SY5Y cells (Pasteur Institute, Iran) were cultured in Dulbecco modified Eagle Medium (DMEM) (Gibco, USA) supplemented with 10% foetal bovine serum (FBS) (Gibco, USA) and 100 U/mL penicillin/streptomycin (Gibco, USA) at a pH of 7.4 and maintained at 37ºC in a humidified atmosphere of 5% CO2 and 95% air. Culture medium was exchanged every 3‐4 days, and the cells were harvested and dispersed at 65%‐75% confluency. MPP^+^ (Sigma, USA) was diluted in medium with low serum to four ultimate concentrations. Twenty‐four hours prior to initiating each experiment, culture medium was changed to 1% FBS. To create an acute toxicity cellular model, SH‐SY5Y cells were exposed to 500, 1000, 2000 and 3000 μmol/L MPP^+^ for 24 hours. Chronic model of toxicity was also produced by MPP^+^ treatment with the same concentrations three times per week for 2 weeks.

### 2.4 Cell viability assessment

Cell viability was monitored by MTS assay, a marker of mitochondrial activity, in which viable cells bioreduce the administered [3‐(4,5‐dimethyl‐thiazol‐2‐yl)‐5‐(3‐carboxymethoxy phenyl)‐2‐(4‐sulfophenyl)‐2H‐tetrazolium, inner salt, MTS to a coloured soluble formazan. First, SH‐SY5Y cells were seeded into plates and treated with various concentrations of neurotoxin. Next, MTS/PMS solution (Promega, USA) was added to each well and incubated for 4 hours at 37°C. Subsequently, the amount of produced formazan was ascertained at a wavelength of 490 nm using the ELISA microplate reader (Awareness, USA). The absorbance values of MPP^+^‐exposed cells were reported as a percentage of the untreated control cultures.

### 2.5 Flow cytometric detection of apoptotic cells

Apoptosis was detected by cytofluorometric analysis using a FACSCalibur (Becton Dickinson, Mountain View, CA, USA). Apoptotic rate induced by chronic or acute MPP^+^ toxicity was quantified using the Phosphatidyl Serine Detection Kit (IQ products‐) based on the manufacturer's recommendations. Briefly, after exposure to MPP^+^, SHSY‐5Y cells were collected, washed and incubated with 10 μL of Annexin V‐FITC at 4°C in the dark for 20 minutes and then, 10 μL of PI. Ultimately, the fluorescence intensity was analysed by FACSCalibur flow cytometer and CellQuest software.

### 2.6 Intracellular ROS measurement

The amount of ROS was estimated using dichlorofluorescein‐diacetate (DCFH‐DA) assay. After crossing the membrane, fluorescent dye DCFH‐DA is enzymatically hydrolysed to non‐fluorescent DCFH which can be oxidized by ROS to 2′,7′‐dichlorofluorescein (DCF), a highly fluorescent compound. First, MPP^+^‐intoxicated SHSY‐5Y cells were incubated at 37°C with diluted DCFH‐DA (Sigma, USA) (0.5 μmol/L) for 20 minutes in dark. Next, the cells were washed with serum‐free media three times to wipe out the dissociative DCFH‐DA. After that, fluorescence intensity of DCF in cell samples was measured by the flow cytometry apparatus.

### 2.7 RNA extraction, synthesis of cDNA and real‐time PCR

Total RNA was extracted from PBMCs and toxin‐treated cells based on manufacturer's instructions by TRIzol reagent (Invitrogen, USA) and dissolved in RNase‐free water. Then, isolated RNAs were treated with DNaseI (Ferments, USA) to eliminate any DNA contamination. To verify the purity of RNA samples, they were examined by NanoDrop spectrometer (Biochrom WPA, Biowave, UK). Additionally, the integrity was assessed by running RNA samples on 1% agarose gel. The miR‐CURY LNA Universal RT microRNA PCR kit (Exiqon, Denmark) was applied to synthesize complementary DNAs (cDNAs) for miR‐376a with 100 ng of RNA. The tubes were incubated at 42°C for 60 minutes, before heat inactivation of reverse transcriptase enzyme at 95°C for 5 minutes. Then, real‐time quantitative PCR was conducted on cDNA product utilizing ExiLENT SYBR Green master mix accompanied by the miRNA‐specific locked nucleic acid (LNA) PCR primer pairs (Exiqon, Denmark) in Step One plus Real‐Time PCR thermal cycler (Applied Biosystems, USA). cDNA synthesis for *PGC1α*, *TFAM* and *GSK3β* genes was carried out on 1 μg of RNA reverse transcribed by cDNA Synthesis Kit (TaKaRa, Japan) by random hexamer primers. Real‐time PCR was conducted in Step One plus Real‐Time PCR thermal cycler using SYBR premix ExTaq II (TaKaRa, Japan) and specific primer sets (Table 1) synthesized by Macrogen Company, South Korea. miRNA and genes’ expression data were normalized to the expression levels of small nucleolar RNA U6 and *glyceraldehyde 3‐phosphate dehydrogenase* (*GAPDH*), respectively. Relative quantification calculations were all assessed according to the comparative Ct (2^−ΔΔCt^) method.

**Table 1:** RT‐qPCR primers for mRNAs

mRNA	Primer name	Primer sequence
*PGC‐1α*	Sense primer	5'‐GACACAACACGGACAGAACT‐3'
	Antisense primer	5'‐GCATCACAGGTATAACGGTAGG‐3'
*TFAM*	Sense primer	5'‐AATAGATAGGATGGGTTTGAG‐3'
	Antisense primer	5'‐AGATGACACAGGGACTTA‐3'
*GSK3β*	Sense primer	5'‐GTGTTCATTCCAGCAAGG‐3'
	Antisense primer	5'‐GCCAGTGTCTTCATATCC‐3'
*GAPDH*	Sense primer	5'‐TGCCGCCTGGAGAAACC‐3'
	Antisense primer	5'‐TGAAGTCGCAGGAGACAACC‐3'

### 2.8 Statistical analysis

All statistical analyses were conducted by SPSS 22.0. and GraphPad Prism 6 software. The disparity between means was analysed by Mann‐Whitney test, Student's independent‐samples *t* test, chi‐square test as well as one‐way ANOVA. The Kolmogorov‐Smirnov normality test (KS‐test) was served to evaluate the normality of data distribution. To examine the diagnostic sensitivity and specificity of miR‐376a level to discriminate between PD cases and healthy subjects, the receiver operating characteristic (ROC) curve was drawn and MEDCALC (http://www.medcalc.org) online software was also recruited to calculate positive predictive value (PPV) and negative predictive value (NPV). Spearman coefficient calculation was also used to analyse possible correlations between parameters of interest. Data are expressed as the mean ± SD of three independent experiments, and *P*‐values <.05 were deemed to be significant differences statistically.

## 3 RESULTS

### 3.1 Characteristics of study participants

Demographic information and clinical information of all subjects are listed in Table 2. Although statistical analyses demonstrated no significant differences in terms of gender and age between two groups of subjects and stages, there was a significant correlation between PD stages and disease duration (*P* = .007).

**Table 2:** Demographic and clinical data of study participants

	Number		Gender		Age (y)	Disease duration (Mo)
			F	M		
Control	25		9	16	60.28 ± 10.125	–
PD	33		10	23	62.904 ± 11.430	69.985 ± 58.856
Early‐stage PD	HY I	12	8	18	60.30 ± 10.227	44.590 ± 29.722
	HY II	14				
Advanced stage PD	HY III	4	2	5	66 ± 12.200	131.657 ± 66.044
	HY IV	3				
	*P‐*value		.647^a^		.667^b^	.007^d^
			.910578^a^		.157^c^	

Note

Data are presented as mean ± SD. Two diagnostic groups and stages had no significant differences in distributions of age and sexuality, while there was a significant correlation between PD stages and disease duration (*P* = .007).

Abbreviations: F, female; M, male; PD, Parkinson's disease; HY, Hoehn and Yahr scales.

a *P*‐values were calculated using chi‐square test.

b *P*‐value was calculated by Student's *t* test.

c *P*‐value was calculated using one‐way ANOVA.

d *P*‐value was calculated recruiting Mann‐Whitney test.

### 3.2 In silico findings

To choose a candidate miRNA, following criteria were considered: (a) strong predicted interactions between the miRNA and candidate genes. (b) Deregulation of the miRNA in various neurodegenerative conditions.

**Figure 1:**
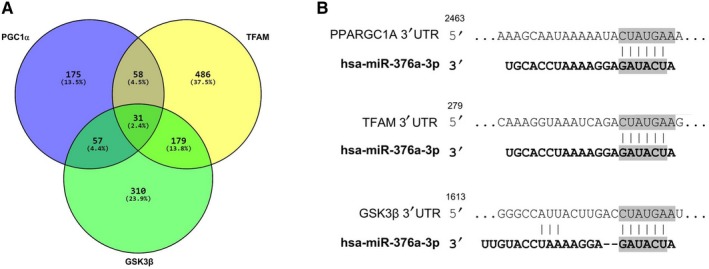
miRNA selection. (A)Venn diagrams were recruited to obtain the common predicted miRNAs which target *PGC1α*, *TFAM* and *GSK3β* mRNAs. Numbers demonstrate the quantity of miRNAs which target the circled mRNAs. Thirty‐one (2.4%) miRNAs were common between three mRNAs and selected for more analyses, (B) the potential matching positions of miR‐376a within the 3′‐UTR of *PGC1α*, *TFAM* and *GSK3β* predicted by TargetScan are depicted in the illustration

First, online prediction software, TargetScan, was recruited to predict the miRNA which target candidate mRNAs; *PGC1α*, *TFAM* and *GSK3β*. To restrict the number of miRNAs, we only considered stronger interactions with context ++ score value more than −0.1. Secondly, the predicted targeting microRNAs of selected genes by prediction programme were then compared by Venn diagrams to detect overlapping miRNAs (Figure 1). We found 31 miRNAs targeting *PGC1α*, *TFAM* and *GSK3β* at once, and prediction information related to miRNA‐mRNA pairs was taken from TargetScan database as shown in Table 3. After that, literature mining was carried out to identify deregulated miRNAs’ profiles in neurodegenerative conditions.

**Table 3:** Prediction information of 31 common miRNAs

miRNAs	mRNA	Position in the UTR	Seed match	context++ score^a^	context++ score percentile
hsa‐miR‐199b‐5p	GSK3B	61‐67	7mer‐m8	−0.22	91
	PPARGC1A	1485‐1492	8mer	−0.39	96
	TFAM	2524‐2531	8mer	−0.2	90
hsa‐miR‐181c‐3p	GSK3B	1178‐1184	7mer‐1A	−0.14	85
	PPARGC1A	1785‐1792	8mer	−0.32	99
	TFAM	6014‐6020	7mer‐1A	−0.19	91
hsa‐miR‐539‐3p	GSK3B	4390‐4397	8mer	−0.16	75
	PPARGC1A	3714‐3721	8mer	−0.27	92
	TFAM	6214‐6220	7mer‐m8	−0.16	74
hsa‐miR‐6842‐5p	GSK3B	2922‐2928	7mer‐m8	−0.21	67
	PPARGC1A	2314‐2321	8mer	−0.25	74
	TFAM	6141‐6147	7mer‐1A	−0.17	56
hsa‐miR‐7110‐5p	GSK3B	2922‐2928	7mer‐m8	−0.28	78
	PPARGC1A	2314‐2321	8mer	−0.22	67
	TFAM	6141‐6147	7mer‐1A	−0.11	40
hsa‐miR‐3124‐3p	GSK3B	76‐82	7mer‐1A	−0.19	93
	PPARGC1A	539‐546	8mer	−0.21	95
	TFAM	3217‐3224	8mer	−0.22	95
hsa‐miR‐6752‐5p	GSK3B	2922‐2928	7mer‐m8	−0.16	65
	PPARGC1A	2314‐2321	8mer	−0.21	76
	TFAM	6141‐6147	7mer‐1A	−0.13	55
hsa‐miR‐4712‐3p	GSK3B	2407‐2413	7mer‐m8	−0.11	81
	PPARGC1A	675‐682	8mer	−0.2	93
	TFAM	3958‐3964	7mer‐m8	−0.18	91
hsa‐miR‐876‐3p	GSK3B	31‐38	8mer	−0.29	94
	PPARGC1A	582‐588	7mer‐m8	−0.19	86
	TFAM	3625‐3631	7mer‐1A	−0.1	70
hsa‐miR‐6773‐3p	GSK3B	3302‐3309	8mer	−0.15	85
	PPARGC1A	3532‐3539	8mer	−0.19	90
	TFAM	3669‐3675	7mer‐m8	−0.14	85
hsa‐miR‐218‐5p	GSK3B	4142‐4148	7mer‐m8	−0.12	77
	PPARGC1A	229‐235	7mer‐m8	−0.17	85
	TFAM	3808‐3814	7mer‐m8	−0.15	83
hsa‐miR‐23c	GSK3B	1001‐1008	8mer	−0.12	82
	PPARGC1A	3550‐3557	8mer	−0.17	88
	TFAM	241‐248	8mer	−0.33	95
hsa‐miR‐130a‐5p	GSK3B	1001‐1008	8mer	−0.13	84
	PPARGC1A	3504‐3510	7mer‐m8	−0.11	80
	TFAM	241‐248	8mer	−0.29	95
hsa‐miR‐23b‐3p	GSK3B	1001‐1008	8mer	−0.12	82
	PPARGC1A	3550‐3557	8mer	−0.15	87
	TFAM	241‐248	8mer	−0.34	96
hsa‐miR‐23a‐3p	GSK3B	1001‐1008	8mer	−0.11	79
	PPARGC1A	3550‐3557	8mer	−0.15	87
	TFAM	241‐248	8mer	−0.33	95
hsa‐miR‐376a‐3p	GSK3B	1613‐1619	7mer‐1A	−0.1	61
	PPARGC1A	2463‐2469	7mer‐1A	−0.15	77
	TFAM	279‐285	7mer‐1A	−0.19	86
hsa‐miR‐376b‐3p	GSK3B	1613‐1619	7mer‐1A	−0.1	61
	PPARGC1A	2463‐2469	7mer‐1A	−0.15	76
	TFAM	279‐285	7mer‐1A	−0.19	86
hsa‐miR‐4728‐3p	GSK3B	2157‐2163	7mer‐1A	−0.1	78
	PPARGC1A	3091‐3098	8mer	−0.15	87
	TFAM	148‐154	7mer‐1A	−0.17	89
hsa‐miR‐3941	GSK3B	1263‐1269	7mer‐m8	−0.24	96
	PPARGC1A	312‐318	7mer‐m8	−0.13	87
	TFAM	4573‐4579	7mer‐1A	−0.1	83
hsa‐miR‐8485	GSK3B	2447‐2453	7mer‐m8	−0.13	80
	PPARGC1A	2180‐2186	7mer‐1A	−0.12	79
	TFAM	800‐806	7mer‐m8	−0.22	94
hsa‐miR‐494‐5p	GSK3B	4284‐4291	8mer	−0.52	98
	PPARGC1A	553‐559	7mer‐1A	−0.13	73
	TFAM	4037‐4043	7mer‐1A	−0.2	85
hsa‐miR‐1260a	GSK3B	2493‐2499	7mer‐m8	−0.16	80
	PPARGC1A	803‐809	7mer‐m8	−0.13	73
	TFAM	5627‐5633	7mer‐m8	−0.21	88
hsa‐miR‐1260b	GSK3B	2493‐2499	7mer‐m8	−0.16	80
	PPARGC1A	803‐809	7mer‐m8	−0.13	73
	TFAM	5627‐5633	7mer‐m8	−0.21	88
hsa‐miR‐603	GSK3B	1263‐1269	7mer‐1A	−0.1	90
	PPARGC1A	2179‐2186	8mer	−0.12	92
	TFAM	407‐413	7mer‐m8	−0.14	94
hsa‐miR‐323b‐5p	GSK3B	4284‐4291	8mer	−0.5	98
	PPARGC1A	553‐559	7mer‐1A	−0.11	68
	TFAM	4037‐4043	7mer‐1A	−0.17	80
hsa‐miR‐410‐5p	GSK3B	4284‐4291	8mer	−0.5	98
	PPARGC1A	553‐559	7mer‐1A	−0.11	67
	TFAM	4037‐4043	7mer‐1A	−0.18	82
hsa‐miR‐144‐5p	GSK3B	4972‐4978	7mer‐1A	−0.1	67
	PPARGC1A	1537‐1543	7mer‐1A	−0.11	70
	TFAM	4925‐4931	7mer‐1A	−0.11	72
hsa‐miR‐4465	GSK3B	4636‐4643	8mer	−0.21	84
	PPARGC1A	659‐665	7mer‐1A	−0.1	53
	TFAM	1008‐1015	8mer	−0.32	95
hsa‐miR‐6829‐3p	GSK3B	701‐707	7mer‐m8	−0.14	88
	PPARGC1A	3080‐3086	7mer‐1A	−0.1	80
	TFAM	343‐349	7mer‐1A	−0.13	86
hsa‐miR‐199a‐5p	GSK3B	61‐67	7mer‐m8	−0.22	91
	PPARGC1A	1485‐1492	8mer	−0.39	97
	TFAM	2524‐2531	8mer	−0.2	90
hsa‐miR‐3617‐3p	GSK3B	377‐383	7mer‐m8	−0.11	69
	PPARGC1A	3559‐3565	7mer‐m8	−0.1	67
	TFAM	5687‐5693	7mer‐1A	−0.15	77

Note

This table represents prediction information related to the interactions of 31 miRNAs with *PGC1α, TFAM* and *GSK3β* mRNAs retrieved from TargetScan database.

a As the most important value shown on the table, the context++ score (CS) for a specific site is the sum of the contribution of 14 crucial features including site type, 3' UTR length, supplementary pairing and local AU.

From the constructed list, 31 miRNAs were chosen and then literature mining was carried out to choose one miRNA with altered expression profiles in various NDDs. Finally, neuron‐enriched miR‐376a was chosen as candidate miRNA because of high probability of its binding to candidate genes according to in silico analyses, and also its altered level in different neurodegenerative disorders comprising AD, GBM, PD, MS and SCA1. The potential binding sites of miR‐376a within *PGC1α*, *TFAM* and *GSK3β* mRNAs’ 3 UTRs predicted by TargetScan are shown in Figure 1B.

### 3.3 Dose‐dependent cell viability loss after MPP^+^ treatment

To evaluate the survival of SHSY‐5Y cells in response to acute oxidative damage, the cells were exposed to various doses of MPP^+^ (500‐3000 μmol/L) for 24 hours. MTS assay showed that MPP^+^ exposure remarkably lessened cell viability in a dose‐dependent manner. Ratio of survival was 58% of that of control for treatment with 2000 μmol/L MPP^+^ for 24 hours, and decreased more to around 41% after exposure to 3000 μmol/L MPP^+^. 2000 μmol/L MPP^+^ was chosen as an optimal concentration for creating acute PD model reducing cell viability by 42%.

**Figure 2:**
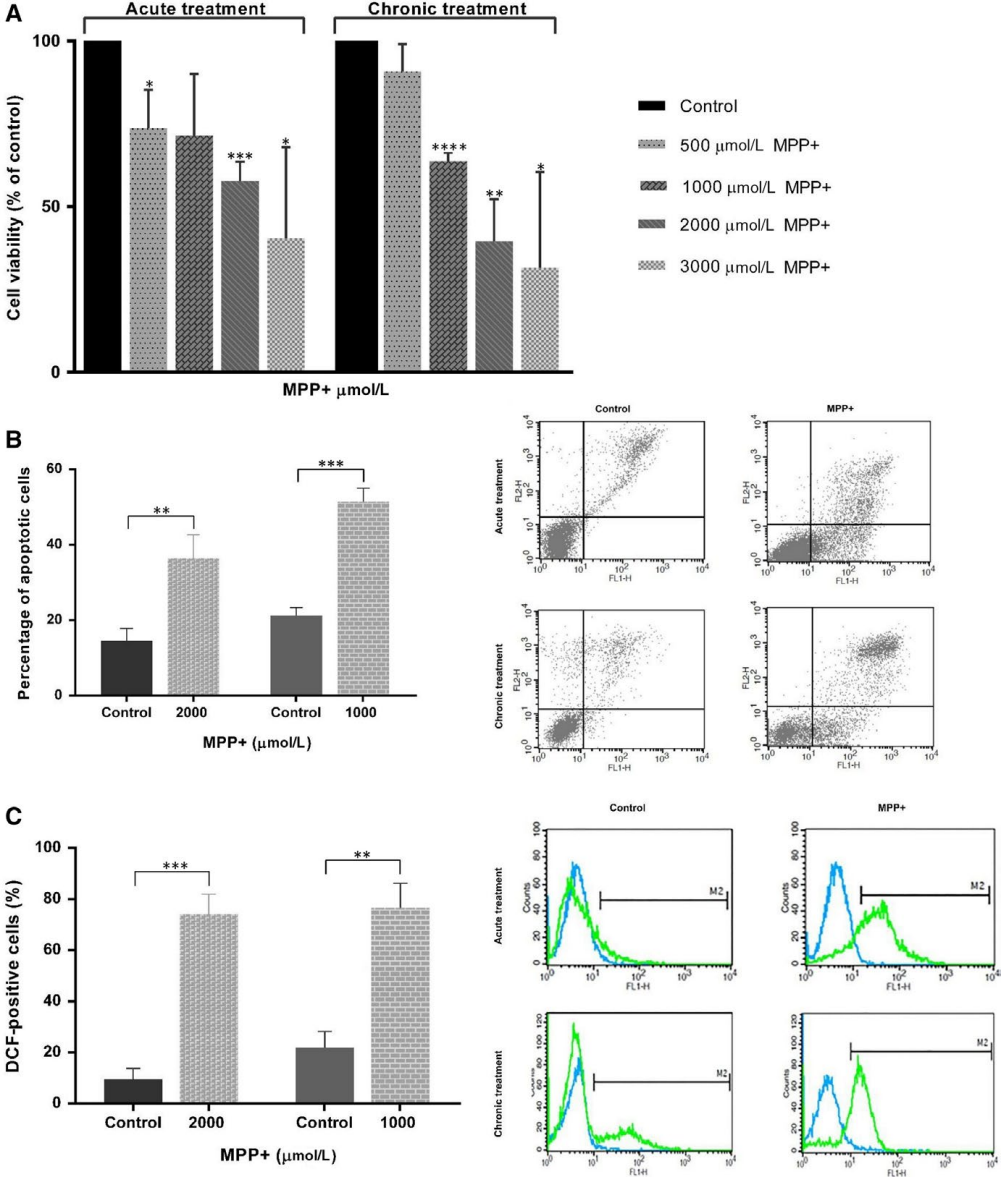
MPP^+^‐induced cell death, apoptosis and intracellular ROS overproduction in SHSY‐5Y cells. (A) SHSY‐5Y cells were exposed to various concentrations of MPP^+^ for 24 h and 2 wk. Cell viability was monitored using MTS assay. Two thousand and 1000 μmol/L MPP^+^ were selected as optimal concentrations for creating acute and chronic PD models because of inducing cell death by 42% and 37%, respectively. (B) Flow cytometric detection of apoptotic cells using Annexin V‐FITC staining. The histogram represents percentages of apoptotic cells in total cells for SHSY‐5Y cells treated with 2000 μmol/L MPP^+^ for 24 hours in comparison with untreated cells (left) and SHSY‐5Y cells exposed to 1000 μmol/L MPP^+^ for 2 wk compared with control cells (right). (C) Flow cytometric analysis of ROS production using DCFH‐DA staining. The histogram demonstrates percentages of DCF‐positive cells in total cells for SHSY‐5Y cells exposed to 2000 μmol/L MPP^+^ for 24 hours compared with control cells (left) and SHSY‐5Y cells treated with 1000 μmol/L MPP^+^ for 2 wk in comparison with untreated cells (right). (*P < .05, **P < .01 and ***P < .001 vs control, independent‐samples *t* test)

Optimal MPP^+^ concentration to create chronic PD model was also selected based on MTS assay. SHSY‐5Y cells were exposed to the same concentrations of MPP^+^ three times per week for up to 2 weeks. According to MTS analysis, 1000 μmol/L MPP^+^ which induced about 37% cell death was chosen as an appropriate concentration to make a chronic cellular model of PD (Figure 2A).

### 3.4 MPP^+^‐induced increase in apoptosis

Rate of apoptotic cell death induced by MPP^+^ was quantified by Annexin V‐FITC Staining. SHSY‐5Y cells were incubated with MPP^+^ acutely and chronically, and apoptotic cells were then recognized by flow cytometry. Most cells were healthy in the control group of acute treatment, and 14% of cells were positive for Annexin V‐FITC binding representing apoptotic cells. Acute MPP^+^ exposure remarkably enhanced apoptotic rate compared with untreated cells. The proportion of apoptotic cells were 37% in SHSY‐5Y cells treated acutely with MPP^+^. Similar to acute model, chronic MPP^+^ exposure caused a marked rise in number of apoptotic cells in comparison with control cells (Figure 2B).

### 3.5 MPP^+^‐induced ROS overproduction

Intracellular ROS formation was assessed by detecting alterations in DCF fluorescence intensity using flow cytometry. To examine MPP^+^ effect on ROS generation and subsequent oxidative stress, SHSY‐5Y cells were exposed to MPP^+^ acutely and chronically for 24 hours and 2 weeks, respectively. In the control group of acute toxicity, the proportion of the cells which were positive for DCF dye was only 10% of total. MPP^+^ treatment caused a dramatic rise in the number of DCF‐positive cells, showing intensified ROS production compared with control group. The proportion of DCF‐positive cells were 74% in SHSY‐5Y cells treated acutely with MPP^+^. Similarly, exposure of SHSY‐5Y cells to chronic MPP^+^ resulted in a considerable growth in the number of DCF‐positive cells compared with untreated cells (Figure 2C).

### 3.6 Down‐regulation of miR‐376a and up‐regulation of genes’ transcript levels after acute MPP^+^ treatment

**Figure 3:**
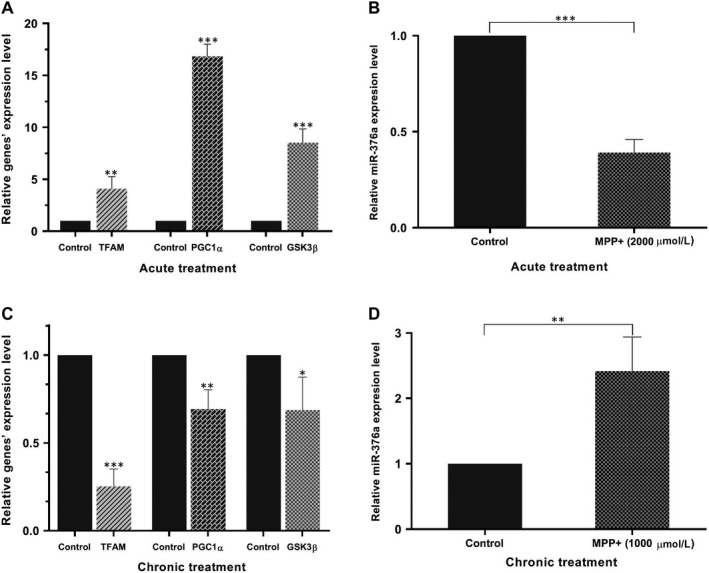
Deregulation of *PGC1α*, *TFAM* and *GSK3β* as well as miR‐376a in acute and chronic MPP^+^‐treated SHSY‐5Y cells. (A) RT‐qPCR analysis of *PGC1α*, *TFAM* and *GSK3β* transcripts expression in acute MPP^+^‐treated and untreated SHSY‐5Y cells. (B) RT‐qPCR analysis of miR‐376a level in acute MPP^+^‐treated and untreated SHSY‐5Y cells. (C) RT‐qPCR analysis of *PGC1α*, *TFAM* and *GSK3β* expressions in chronic MPP^+^‐treated and untreated SHSY‐5Y cells. (D) RT‐qPCR analysis of miR‐376a expression in chronic MPP^+^‐treated and untreated SHSY‐5Y cells. Transcript levels were normalized to the expression level of *GAPDH* as the reference gene, and miRNA level was normalized to expression level of the U6 snRNA as the reference gene. (*P < .05, **P < .01 and ***P < .001, vs. control, independent‐samples *t* test)

In order to evaluate alterations in candidate genes’ expression, RT‐qPCR was performed, representing that *TFAM*, *GSK3β* and particularly, *PGC1α* levels were significantly affected by acute oxidative damage. The transcript levels of *PGC1α* markedly raised following treatment with 2000 μmol/L MPP^+^ for 24 hours, in comparison with that of unexposed control. Similarly, *TFAM* and *GSK3β* were substantially up‐regulated following exposure to MPP^+^ (Figure 3A). The expression levels of candidate genes were normalized to *GAPDH* as a reference gene. Alteration in miR‐376a level was also quantified by real‐time PCR analysis, and expression was normalized to U6 snRNA that has been found to be an appropriate internal control for miRNAs’ studies in SHSY‐5Y cells. Exposing SHSY‐5Y cells to 2000 μmol/L MPP^+^ for 24 hours culminated in a dramatic drop in miR‐376a level in comparison with untreated cells as a control group (Figure 3B).

### 3.7 Up‐regulation of miR‐376a and down‐regulation of genes’ mRNA levels in response to chronic MPP^+^ treatment

In spite of acute MPP^+^ model, levels of *PGC1α*, *TFAM* and *GSK3β* transcripts dropped dramatically in SHSY‐5Y cells chronically stressed with MPP^+^ (Figure 3C). Moreover, low dose, repeated 1000 μmol/L MPP^+^ administration caused a significant growth in miR‐376a level contrary to acute toxicity (Figure 3D).

### 3.8 Up‐regulation of miR‐376a and down‐regulation of genes’ mRNA levels in PBMCs derived from PD patients

**Figure 4:**
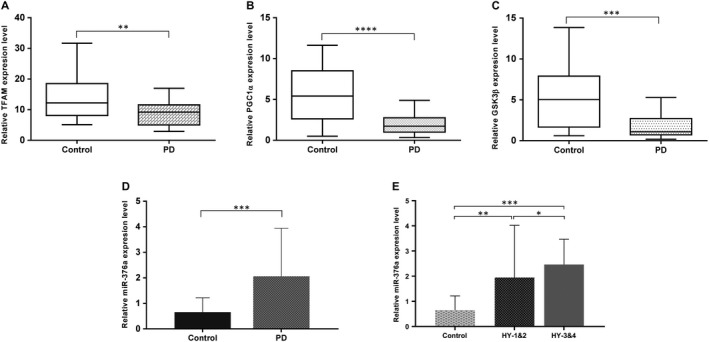
Down‐regulation of *TFAM, PGC1α*  and *GSK3β* and up‐regulation of miR‐376a in PBMCs derived from PD patients. RT‐qPCR analysis of (A)*TFAM*, (B) *PGC1α* and (C) *GSK3β* transcripts in PBMCs from PD patients compared with age‐matched healthy controls. (D) RT‐qPCR analysis of miR‐376a expression in PD PBMCs compared with healthy controls. (E) Statistical analysis showing the increase in miR‐376a expression with disease severity. (**P < .01, ***P < .001 vs control, independent‐samples *t* test)

PBMCs of 33 PD subjects and 25 age‐matched controls were assessed by measuring the mRNA levels of three mitochondria‐related genes. As depicted in Figure 4A, A statistically dramatic drop in both *PGC1α* and *TFAM* levels occurred in PD patients compared with age‐matched controls. Additionally, PD PBMCs showed a marked decline in *GSK3β* mRNA expression compared with healthy controls. Our results also represented that miR‐376a was strongly up‐regulated in PD patient's PBMCs (Figure 4B).

### 3.9 miR‐376a expression in PD PBMCs is correlated with disease severity

In order to gain more insight into the connection between the progression of PD and miR‐376a level, statistical analysis was performed showing that the expression of miR‐376a in advanced stages is significantly higher than that in stages 1 and 2 of PD and increases with disease severity (Figure 4C).

### 3.10 Diagnostic significance of miR‐3 6a expression

**Figure 5:**
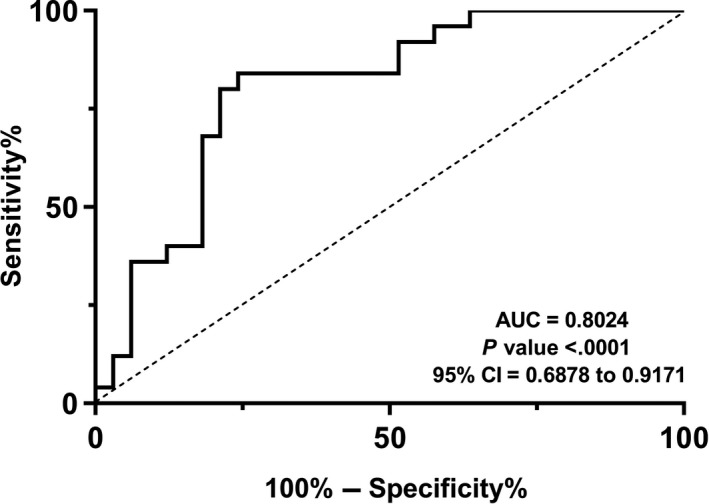
Diagnostic significance of miR‐376a expression. ROC curve analysis performed to examine the sensitivity and specificity of miR‐376a expressions for PD diagnosis calculated a high significant capability of miR‐376a expression level as a diagnostic biomarker to distinguish PD patients from healthy controls. (AUC = 0.8024, *P* < .0001. 95% CI = 0. 6878 to 0.9171)

The suitability of miR‐376a to distinguish PD patients from healthy controls was investigated by performing ROC analysis. Higher total area under the curve (AUC) value demonstrates more effective overall performance of the diagnostic marker to accurately discriminate between certain two conditions. Hence, total are under the ROC curve represents the fact that miR‐376a level (AUC = 0.8024, *P* < .0001. 95% CI = 0.6878 to 0.9171) may serve as a biomarker for discriminating between PD cases and normal controls (Figure 5A). Notably, miR‐376a also had relatively high NPV (72.41%, 95% CI = 58.37% to 83.09%) and PPV (86.21%, 95% CI = 71.38% to 94.00%), showing diagnostic value of this miRNA in PD.

### 3.11 Decreased expression of PGC1α and TFAM negatively correlates with miR‐376a expression in PD PBMCs

According to opposing patterns of miR‐376a and three candidate genes in cellular models, we hypothesized that altered miR‐376a expression in PBMC may be involved in the aberrant mRNA levels in PD patients. To test this hypothesis in the latter, the correlations between the level of miR‐376a and predicted target genes, *PGC1α*, *TFAM* and *GSK3β*, were assessed via statistical analysis. Significant inverse correlations between miR‐376a and *PGC1α* and *TFAM* levels were indicated by Spearman's correlation test (*r* = −.5321 and *P* = .0014, *r* = −.5424 and *P* = .0011, respectively). However, there was no significant correlation between *GSK3β* and miR‐376a levels (*P* = .0578) (data not shown).

## 4 DISCUSSION

While a large number of resources have been allocated to investigate the pathomechanisms of PD in recent years, the disease is still a major challenge in neurobiology since there is no definitive cure for it currently.^5^, ^10^ Progress in PD research has been limited by low availability of targeted brain tissue and reliance on post‐mortem samples. Thus, several cellular and animal PD models are developed and widely used as alternatives for these difficult‐to‐access tissues. Given that most of the studied cellular and animal models are acute, in addition to acute model, we employed a chronic neurotoxin‐induced in vitro model of PD in which degeneration develops during a protracted period and characteristics of PD pathogenesis such as oxidative stress and modest complex I defect are reproduced.^19^ PBMC can be used as a non‐invasive peripheral laboratory sample to study molecular signatures of NDDs that could support their diagnosis.^20^ Especially, in silico analyses represent similar modifications for distinct factors may happen in brain and PBMC during the progress of NDDs in which introduce the PBMC as an appropriate sample to monitor these diseases.^20^, ^21^ Although a wide variety of studies use patients’ sera to examine non‐coding RNAs profiles, their sources should be further clarified. However, similarities in transcript patterns of a number of such factors, in both PBMC and brain in PD, provide us a reason to assess such factors in PBMC.^22^ Accordingly, we investigated gene expression's differences in both acute and chronic in vitro PD models as well as PBMCs of patients with PD.

Although the root cause of sporadic PD is intricate and elusive, multiple lines of evidence associate its pathogenesis to mitochondrial dysfunction.^2^ As a common feature of PD, mitochondrial dysfunction decreases complex I activity, intensifies production of oxygen radicals and causes abnormal protein‐protein interactions, leading to cell death.^6^, ^10^ Thus, MPTP and its active metabolite, MPP^+^, as inhibitors of complex I of mitochondrial respiratory chain have been widely applied to create various models of PD and to study molecular mechanisms involved in neurodegeneration.^2^, ^5^ The principal mechanism of MPP^+^ toxicity is through complex I inhibition followed by mitochondrial dysfunction and oxidative stress, leading to apoptotic cell death.^5^ Consistently, in the current study, ROS generation and apoptotic rate significantly increased after 24‐hours treatment with 2000 μmol/L MPP^+^ (65% and 23% higher than corresponding controls, respectively) and 2 weeks of treatment with 1000 μmol/L MPP^+^ (55% and 30% higher than corresponding controls, respectively), indicating the efficient blockage of complex I by MPP^+^ in SHSY‐5Y cells. There are many remarkable differences between acute and chronic PD models. For instance, in terms of mitochondrial function, acute toxin treatment damages a large proportion of mitochondria and damages go beyond the cell ability to regenerate or repair mitochondria. By contrast, the turnover rate of impaired mitochondria can be more compatible with mitochondrial biogenesis in the chronic MPP^+^ model.^7^ Although the intensity of induced oxidative damage and apoptosis was approximately the same in acute and chronic treatment after 24 hours and 2 weeks of toxin treatment, respectively, our findings confirmed remarkable mechanistic differences between acute and chronic MPP^+^ toxicity, in terms of miRNA and other genes’ expressions patterns, in line with previous studies.^7^

PGC1α is a master regulator of mammalian mitochondrial biogenesis in response to disparate physiological or pathological stresses and deemed to be a valuable potential therapeutic target for PD.^9^, ^10^ PGC1α plays an indispensable role in normal function of mitochondria, and its deficiency can participate in neurodegenerative processes. On top of that, its overexpression was proven to be neuroprotective against neurotoxins in several models.^1^ Numerous studies demonstrated that PGC1α expression can be induced in neurons exposed to oxidative stress.^4^ Totally, studies on changes in PGC1α levels have provided inconsistent data in different PD models. One study showed that PGC1α mRNA was up‐regulated in SH‐SY5Y cells in response to acute MPP^+^ treatment for 24 hours, closely mirroring our result.^10^ Torok et al also reported that the PGC1α mRNA levels were increased in the brain of an acute MPTP‐intoxicated mouse model of PD.^1^ However, PGC1α level has been found to be diminished in SHSY‐5Y cells exposed to MPP^+^ for 48 hours.^8^

*TFAM* knockout mice show declined mtDNA expression and respiratory chain impairments in DA neurons of midbrain, resulting in a parkinsonism phenotype with progressive motor function impairment,^23^ whereas *TFAM* overexpression increases mitochondrial gene expression and respiratory chain proteins.^24^ Furthermore, several studies have reported that overexpressed *TFAM* can reverse the MPP^+^‐mediated reduction in proteins of mitochondrial respiratory complex and successfully ameliorate MPP^+^‐induced mitochondrial dysfunction, verifying the potential significance of TFAM in chronic MPP^+^ toxicity.^2^, ^7^ Consistent with our results, several studies previously have shown that TFAM is reactive to cellular oxidative damage and can be induced by ROS in mammalian cells.^25^ Suliman et al have shown that oxidative damage to mitochondria induced by LPS led to increased mitochondrial TFAM levels.^26^ Additionally, six various LCL cell lines have shown remarkable *TFAM* overexpression compared with human PBMCs, accompanied by increased *TFAM* copy number and protein levels.^25^ This study also confirmed a strong association between intracellular ROS levels and *TFAM* gene expression in LCLs, suggesting that up‐regulation of TFAM may be a cellular defence mechanism to stabilize and to protect mtDNA against ROS‐induced oxidative stress during transformation process of LCLs.^25^ In contrast, *TFAM* level has been reported to diminish in SH‐SY5Y cells following MPP^+^ treatment for 48 hours.^2^ Moreover, some studies have shown that acute MPP^+^ and MPTP suppress expression of *TFAM* in SHSY‐5Y cells and mice brain, respectively.^2^

It is well documented that GSK3β is a crucial pathogenic protein kinase for PD and makes a contribution in the regulation of neuronal apoptosis.^12^ Additionally, some studies carried out in vitro indicated the implication of GSK3β in MPP^+^‐mediated toxicity and mitochondrial dysfunction.^27^ Zhang et al^12^ observed enhanced expression of *GSK3β* at protein and mRNA levels in MPP^+^‐treated SH‐SY5Y cells in concentration‐ and time‐dependent manner. Similarly, we detected dramatically up‐regulated levels of *GSK3β* in response to acute MPP^+^ toxicity. In contrast, total amount of GSK3β did not appear to be affected in MPTP/MPP^+^‐treated neurons.^27^ Collectively, the elevated levels of these genes after acute treatment can be regarded as a short‐term compensatory mechanism against MPP^+^‐induced mitochondrial damage.^1^

It should be noted that only a few studies have assessed changes in genes’ expression in chronic cellular PD model so far. Additionally, although a declined mitochondrial pool of PGC1α was observed in chronic MPP^+^ toxicity, the total protein level of PGC1α was not affected. Chronic toxicity also suppressed *PGC1α* mRNA levels; however, it was not statically significant.^7^ According to Zhu et al, low dose, repetitive MPP^+^ treatment suppressed the expression of *TFAM* remarkably in SHSY‐5Y cells, consistent with our finding.^7^ To our knowledge, there has been no study done on the effect of chronic neurotoxicity on *GSK3β* levels. Taken together, here, the chronic MPP^+^ treatment caused a remarkable reduction in levels of *PGC1α*, *TFAM* and *GSK3β* in SHSY‐5Y cells and this reduction may show an adaptation of cells to chronic neuronal injury evoked by repeated MPP^+^ administration.^1^

In order to evaluate possible roles of candidate genes in mitochondrial impairment in PD blood cells, we also examined the expression levels of *PGC1α*, *TFAM* and *GSK3β* in PD patient's PBMCs. Similar to the chronic PD model, the expression levels of aforementioned genes also decreased in PBMCs of PD patients and this reduction may represent an adaptive mechanism of patient's body to the neurodegenerative condition.^1^ Consistent with our result, as reported by Delbarba et al, AD PBMCs show a significant decrease in *PGC1α* expression, while *PCG1α* was unchanged in mild cognitive impairment (MCI) patients. Additionally, the expression of *TFAM* dropped in PBMCs derived from both AD and MCI patients.^11^ In addition, to the best of our knowledge, this is the first study reporting alterations in *PGC1α*, and *TFAM* levels in peripheral cells of parkinsonian patients. In spite of GSK3β’s central role in PD, only one study has been ever published investigating changes in GSK3β level in PD PBMCs. While the previous study demonstrated enhanced peripheral expression of *GSK3β* in PD patients, the present data show a significant reduction in *GSK3β* levels in PD PBMCs.^28^ Moreover, Marksteiner et al indicated remarkable decrease in GSK3β levels in PBMCs of patients with MCI and AD, which is consistent with our result.^29^ Collectively, our results confirmed the peripheral mitochondria dysfunction in PD and suggest that *PGC1α*, *TFAM* and *GSK3β* down‐regulated levels could be demonstrative of events taking place in PD pathogenesis. However, additional experiments are clearly needed to clarify whether *PGC1α*, *TFAM* and *GSK3β* are appropriate biological markers for PD.

Accumulating evidences are available linking disturbed expression of specific miRNAs to the pathogenesis of NDDs such as PD.^5^, ^15^, ^30^ Indeed, a large number of miRNAs contribute to the direct or indirect modulation of PD through transcriptional regulation of disease‐linked genes. In the current study, miR‐376a was finally chosen as a predicted modulator of the aforementioned genes, through online prediction software as well as literature mining. In the present study, the expression of miR‐376a decreased in acute model, while there was dramatically up‐regulated level of the miRNA in chronic model of PD. To our knowledge, only one study has assessed miR‐376a expression in a PD model, representing that thirty‐minute exposure to 6‐OHDA, followed by 24‐hours recovery, dramatically reduced miR‐376a level in MN9D cells.^31^ Using PBMC as a model of PD, we examined if peripheral expression of miR‐376a is altered in this condition. As miR‐376a is one of the most highly expressed miRNAs in elderly human blood mononuclear cells, aberrant levels of this miRNA may strongly affect its target genes and molecular networks normally controlled by this miRNA in PBMCs.^32^ Therefore, we aimed here to determine miR‐376a alteration in PBMCs from patients with PD compared to healthy subject and found profound increase in miR‐376a expression in PD PBMC samples relative to healthy controls. Up‐regulated level of miR‐376a was also detected in T cells of MS patients.^13^ miR‐376a has been identified to be down‐regulated in the brains of late‐onset form of Alzheimer's disease (LOAD).^14^ Altered abundance of miR‐376a was also reported in other neurodegenerative conditions such as spinocerebellar ataxia type 1 (SCA1) and prion disease.^13^, ^33^

When correlating the miRNA expression with the indices of disorder severity, interestingly, we found out that miR‐376a expression was significantly higher in stage III/IV PD patients than in stage I/II patients and was strongly correlated with disease severity.

Evaluating the possible association of miR‐376a with predicted target genes, *PGC1α*, *TFAM* and *GSK3β*, was the following stage to proceed in the current study. Opposing expression pattern of the miR‐376a with the genes in 3 assessed neurodegenerative conditions was regarded as a validating evidence of predicted interactions. In addition, the significance of the associations between miR‐376a and *PGC1α* and *TFAM* was further validated by Spearman's correlation coefficient data analysis (*r* = −.5321 and *P* = .0014, *r* = −.5424 and *P* = .0011, respectively), meaning that miR‐376a may affect mitochondrial function in PBMCs by modulating *PGC1α* and *TFAM* expression.

To evaluate the overall modulation of miR‐376a in discriminating between PD patients and controls, sensitivity, specificity, NPV and PPV were calculated. Accordingly, AUC in ROC analysis revealed high sensitivity and specificity of miR‐376a in terms of PD patients’ distinction from healthy individuals, demonstrating the possible involvement of miR‐376a in PD, while the mechanisms by which miR‐376a may contribute to PD development await to be precisely identified. Notably, single miRNA expression profile change might not demonstrate robust PD classifiers across various PD subject cohorts, while combinatory miRNA signatures represent more promising complementary diagnostic tools for this disorder. Thus, it is imperative that our future studies evaluate the diagnostic potential of miR‐376a accompanied by other candidates as a miR combination acting as a good classifier across different patient cohort studies.^34^

Collectively, the same expression patterns of miR‐376a and genes in chronic model and PD PBMCs suggest that possibly low‐dose chronic toxicity model can more closely exhibit chronically developing PD than high‐dose acute MPP^+^ models.^35^ The current study also revealed that acute severe mitochondrial dysfunction in SHSY‐5Y cells induced protection via enhancing the expression of *PGC1α*, *TFAM* and *GSK3β*. However, low‐dose chronic MPP^+^ treatment did not induce those mechanisms and instead suppressed expression of genes. Similarly, the expression levels of *PGC1α*, *TFAM* and *GSK3β* decreased in PBMCs of PD patients indicating that brain mitochondrial dysfunction is also reflected in PD PBMCs. Given that miR‐376a and possible target genes showed opposite expression patterns in 3 assessed neurodegenerative conditions and negative correlations were observed between miR‐376a and mRNA levels in PBMCs, we can suggest that miR‐376a may be implicated in the pathogenesis of PD possibly by regulating expression of mitochondria‐related genes.

However, further studies have to be performed to better understand the mechanism enabling this miRNA to operate on its targets in PD. Notably, our findings revealed that overexpression of miR‐376a in PBMCs might be considered as a potential marker of the diagnosis of PD. However, larger‐scale studies using brain samples from PD patients along with independent additional cohort studies must be conducted to confirm the diagnostic significance of this miR.

## CONFLICT OF INTEREST

The authors declare no conflict of interest. All authors support submission to this journal.

## AUTHOR CONTRIBUTIONS

M. B. and M. RD designed the experiments, drafted sections of the manuscript, and performed in silico study, cell culture and real‐time PCR analyses. E. Y. performed flow cytometry. M. P. edited the revised version and incorporated at the final stage of manuscript preparation and data analyses. D. P., M. H. N.‐E and K. G. were responsible for the supervision of project and wrote the manuscript and approved the final version of manuscript. All authors read and approved the final version of manuscript.

## Data Availability

The data that support the findings of this study are available from the corresponding author upon reasonable request.
